# Liver transplantation for HCC within and beyond Milan Criteria: single center experience with literature review

**DOI:** 10.3389/fsurg.2025.1594361

**Published:** 2025-07-10

**Authors:** İ. Tırnova, T. Kanmaz

**Affiliations:** ^1^ Organ Transplantation Center, Koç University Hospital, Istanbul, Türkiye; ^2^ Department of Surgery, Division of Organ Transplantation, Başkent University, Istanbul Hospital, Istanbul, Türkiye

**Keywords:** liver transplantation, Milan Criteria, extended criteria, hepatocellular carcinoma, HCC

## Abstract

**Introduction:**

The Milan Criteria (MC) are widely accepted as the standard patient selection criteria for liver transplantation (LT). However, patients who exceed these criteria may still benefit from transplantation. Various extended criteria have been published. This study aimed to evaluate survival outcomes in hepatocellular carcinoma (HCC) patients undergoing LT, comparing those within and beyond MC, and to review the role of extended criteria in LT.

**Methods:**

This retrospective, single-center study included adult patients who underwent LT at Koç University between 2018 and 2024. Pathological data were used to categorize patients into two groups: those within MC and beyond MC. Preoperative data and postoperative overall and disease-free survival rates were compared between these groups. Additionally, a comprehensive literature review of studies evaluating extended criteria for LT in HCC patients was conducted.

**Results:**

A total of 45 adult patients were included in the analysis. There were 23(51.1%) patients within MC, and 22(48.9%) patients in the beyond MC group. Demographics, donor types, graft types, tumor differentiations, Child scores, MELD scores, ischemia times, length of intensive care unit stays, length of hospital stays, and mortality rates were similar (*p* > 0.05). Tumor count, total tumor diameter, and microvascular invasion rates were statistically higher in patients beyond MC (*p* < 0.05). Survival analyses revealed no statistically significant differences in 1-year, 3-year, and 5-year survival rates of the patients (*p* > 0.05).

**Conclusion:**

This study highlights the potential for liver transplantation in HCC patients exceeding the Milan Criteria, with survival outcomes comparable to those within the Milan Criteria in certain cases. Despite numerous studies in the literature, optimal criteria for LT patient selection in beyond MC HCC have not been established. An optimal guideline that will help to better understand tumor behavior, guide the decision-making and timing of liver transplantation (LT), and ultimately improve post-transplant outcomes remains a key objective for future research.

## 1 Introduction

Hepatocellular carcinoma (HCC) is makes up the majority of liver-derived malignant tumors (80%) and is the third most common cause of cancer-related death worldwide (1). Liver resection (LR) and liver transplantation (LT) are the two curative surgical options for the treatment of HCC. LT outperforms LR in treating HCC according to long-term survival rates (2). Liver transplantation (LT) can cure both HCC and underlying liver diseases. However, the first papers reported poor survival and high recurrence rates until Mazzaferro's outstanding, epoch-making report in 1996 (3). Mazzafero and colleagues have proposed the well-known Milan Criteria (MC) to select the candidates for liver transplantation according to these criteria; a single HCC nodule that does not exceed 5 cm in diameter or up to three nodules that do not exceed 3 cm in diameter, without vascular invasion or extrahepatic metastasis (4). The 4-year survival rate was 75%, which was remarkable for its time. Since its publication in 1996, the Milan Criteria have remained the cornerstone for liver transplantation worldwide.

Despite the MC's effectiveness in improving LT outcomes, the demand of the individuals on waiting lists had not been adequately met due to organ shortage. The MC limitations and shortage of organ presentation for deceased donor liver transplantation (DDLT) have restricted the successful outcomes obtained by the MC for LT. Moreover, some studies have shown that approximately 25% of the patients within MC (wMC), according to preoperative imaging techniques, had pathological results that took the patients beyond the MC (bMC) (5). Since the publication of MC with it's attractive outcomes, many other centers have developed numerous extended criteria. Nevertheless, MC remains important since 1996 and continues to be included in many current liver transplantation guidelines worldwide (6–10).

This paper aims to review the current literature related to liver transplantation outcomes for hepatocellular carcinoma within and beyond Milan Criteria and to analyse the Koç University Organ Transplant Center's outcomes on LT for hepatocellular carcinoma retrospectively.

## 2 History

Following the declaration of the Milan Criteria, novel extended criteria for LT have been reported from many centers. Some extended criteria are purely morphological, whilst others combine some biological parameters along with the morphological findings.

### 2.1 Morphological criteria

Mazzaferro and colleagues published their trial in 1996 and revealed a remarkable survival benefit for the patient within their criteria (Table 1) (4). It remains the reference for novel criteria based on the tumor burden, which are currently being developed. In chronological order, right after MC, Llovet and colleagues published their trial known as the **Barcelona Criteria** (11). They have selected the candidates for transplant, with a single tumor ≤7 cm, or 3 tumors ≤5 cm, or 5 tumors ≤3 cm. The 5-year overall survival rates were over 50%.

**Table 1:** The Milan Criteria and the summary of expanded criteria for liver transplantation.

Publication	Year	Morphologic Criteria	Other Criteria	Reference
Milan	1996	Single tumor ≤ 5 cm or 3 tumors all ≤ 3 cm	-	(40)
Barcelona	1999	Single tumor ≤ 7 cm or 3 tumors ≤ 5 cm or 5 tumors ≤ 3 cm	-	(11)
UCSF	2001	Single tumor ≤ 6.5 cm or 2-3 tumors ≤ 4.5 cm and TTS ≤ 8 cm	-	(12)
Tokyo	2007	Tumor count ≤ 5, MTD ≤ 5 cm	-	(13)
Berlin	2007	No limit on tumor count, MTD ≤ 6 cm and TTD ≤ 15 cm	-	(14)
Up-to-7	2009	The sum of the size (in cm) of the largest tumor and the count of tumor ≤ 7	No microvascular invasion	(15)
Toronto	2016	No limit in size and count of tumor,	No vascular invasion, no cancer related symptoms, > 8 cm; biopsy of the largest tumor not poorly differentiated	(16)
Hangzhou	2008	TTD ≤ 8 cm; or TTD > 8 cm, well and moderate differentiation	AFP ≤ 100 ng/ml	(17)
French AFP Model	2012	Score ≤ 2 (Tumor diameter varies 1–3 and ≥4)	AFP	(18)
TTV-AFP	2015	TTV < 115 cm^3^	AFP ≤ 400 ng/ml	(19)
Kyoto	2007	Tumor count ≤ 10, MTD ≤ 5 cm	PIVKA II ≤ 400 mAU/ml	(23)

UCSF, university of California, San Francisco; TTD, total tumor diameter; MTD, maximum tumor diameter; TTV: total tumor volume; HALT-HCC: hazard associated with liver transplantation for hepatocellular carcinoma; LiTES: liver transplant expected survival.

Two years after the Barcelona Criteria, Yao and colleagues published the University of California, **San Francisco (UCSF) Criteria** in 2001 (12). Their criteria include patients with a single tumor ≤6.5 cm or ≤3 lesions with the largest one ≤4.5 cm, and total tumor diameter ≤8 cm for LT. Their trial revealed 73% of 5-year overall survival (OS) and 81% of 5-year disease-free survival (DFS) (12).

In 2007, Sugawara et al. published the **Tokyo University** series (13). Up to 5 nodules with a maximum tumor size of ≤5 cm in diameter were considered acceptable candidates for living donor liver transplantation (LDLT) in their study. 1-year and 3-year survival rates were completely similar to those within MC; 97% and 94%, respectively. In the same year, 2007, Jonas et al. from **Germany** published their extended criteria outcomes (14). Multiple HCC nodes were considered acceptable up to a diameter of the largest node of 6 cm and a total tumor diameter (TTD) of 15 cm for their study. Rates of 3-year survival and recurrence-free survival were 68% and 64%, respectively.

Mazzaferro and colleagues published their extended criteria “**Up-to7”** in 2009 (15). Their criteria include the size and number of the tumors. The results were obtained from a retrospective analysis of 1,556 LT patients, showing a group of patients with an estimated 5-year OS in the absence of microvascular invasion (MiVI); seven being the result of the sum of size (in cm) and number of tumors for any given HCC. This included all combinations of a given HCC from one nodule up to 6 cm in size (1 + 6) to many tumors fulfilling seven as the sum of size plus number (2 tumors up to 5 cm, 3 tumors up to 4 cm, 4 tumors up to 3 cm, 5 tumors up to 2 cm). This group of patients had 71.2% 5-year OS rates similar to outcomes of patients wMC. On the other hand, for patients with MiVI, the 5-year overall survival rates decreased to 48.1%. In the analysis for the microvascular invasion rates, the estimated tumor size for no microvascular invasion was 30 mm and 45 mm for patients with microvascular invasion (*p* < 0.001). This result has proposed a possible relation between tumor size and the presence of MiVI.

DuBay and colleagues from the University of Toronto published the extended **Toronto Criteria** in 2011 (16). The Toronto criteria do not limit the size or number of the tumor, but it should not have any vascular invasion, extrahepatic disease, or cancer-related symptoms, and the biopsy can not be poorly differentiated. The 5-year survival outcomes revealed 68% rates.

### 2.2 Combined criteria; AFP

Xu and colleagues revealed the **Hangzhou Criteria** in 2008 from China (17). They reviewed a total of 6,012 HCC patients; of those, 1,352 cases were bMC but within the extended Hangzhou Criteria. Their criteria were: total tumor diameter (TTD) ≤8 cm; or TTD >8 cm, well and moderate differentiation, and preoperative AFP level ≤100 ng/ml. The reported 1-year, 3-year, and 5-year OS rates were 83.1%, 67%, and 59.8%, respectively. They also had a second study group in their trial with AFP levels of 100–400 ng/ml and the same tumor size. This group of patients had worse overall and DFS rates. This demonstrates the effect of AFP level on predicting the prognosis of LT.

Duvuox and colleagues published the **French-AFP** model in 2012 (18). The tumor diameters were divided into three groups: ≪3cm, 3–6 cm, >6 cm, and the number of tumors was divided into two groups: 1–3, and ≥4, in the model. The AFP values were also divided into three groups: <100, 100–1,000, and >1,000. The risk assessment was calculated by the points, varying from zero to four. In this study, it was claimed that the French-AFP model could achieve comparable or even better results than MC in predicting recurrence and survival times. It has also been interpreted that increased AFP levels are associated with vascular invasion and loss of differentiation and are a good tool for predicting tumor behavior. Moreover, it has been stated that AFP provides better prognostic information than tumor diameter and number (18).

Toso and colleagues investigated 6,478 patients who underwent LT and published their extended criteria as “total tumor volume and AFP” (**TTV-AFP**) criteria proposed the following rules: tumor volume <115 cm^3^ and AFP levels under 400 ng/ml in 2015. They presented 75% of 4-year OS rates for the patients within their criteria (19). Toso et al. have already published the “Total tumor volume” criteria in 2008, and they determined the cut-off values of the tumor volumes from their previous study (20).

**The Metroticket 2.0** model includes AFP levels in the algorithm, showing an individualized increase in the risk of death following LT proportional to increased AFP levels (21). Nine years after the publication of the Up-to-7 criteria, Mazzaferro and colleagues published the Metroticket 2.0 in 2018. By the combination of Italian and Chinese data with 1,359 patients, Mazzaferro and colleagues aimed to determine the risk factors affecting the survival rates of patients with HCC. In addition to the Up-to-7 criteria, they prompted AFP levels as a risk factor in their trial. The Milan Criteria's rate of 70% for 5-year overall survival led to determining the extended criteria for Metroticket. In conclusion, they recommended performing LT for patients with HCC with the following features; AFP should be <200 ng/ml and the sum of the number and size of tumors (in centimeters) should not exceed 7; if the level of AFP 200–400 ng/ml, the sum of the number and size of tumors should be ≤5; if the level of AFP 400–1,000 ng/ml, the sum of the number and size of tumors should be ≤4.

The Japanese Liver Transplantation Society proposed the **5-5-500 Model** for LT candidates in 2019 (22). The model includes patients with the number of lesions <5, tumor size ≤5 cm, and AFP levels ≤500 ng/ml. Their results proposed a 19% increase in patients to be candidates for LT when compared with MC. The new criteria provided the 7.3% 5-year recurrence rate and +70% 5-year OS rate.

### 2.3 Combined criteria; alternatives and additions to AFP

Kaido and colleagues published the current **Kyoto Criteria** results (23). Their criteria involve a combination of tumor number ≤10, maximal diameter of each tumor ≤5 cm, and serum des-gamma-carboxy prothrombin (DCP) levels ≤400 mAU/ml. 198 HCC patients were enrolled in the study. 147 of them were within the Kyoto criteria, and 49 of them exceeded the criteria. The 5-year OS rates were 82% and 42%, respectively. The 5-year recurrence rates were 4% and 51%, respectively. The 5-year overall survival rates were similar for both wMC (*n* = 118) and bMC (*n* = 80) groups. The patients were divided into three groups: wMC/Kyoto in (*n* = 105), bMC/Kyoto in (*n* = 42), and bMC/Kyoto out (*n* = 36). The overall survival rates were similar in the wMC/Kyoto in and the bMC/Kyoto in groups. The bMC/Kyoto out group had the worst 5-year survival rates. Also, the same group had the worst 5-year DFS rates.

In 2016, Lee and colleagues developed a model to predict tumor recurrence after LDLT for HCC, with the well-known name, **MoRAL** (24). They used the serum AFP and serum protein induced by vitamin K absence-II (PIVKA-II) levels to calculate risk stratifications for HCC recurrence. They showed that the serum AFP levels were significantly associated with the maximal tumor size. In this study, serum AFP and serum PIVKA levels were used to calculate the MoRAL score. The higher MoRAL score (>314,8) has been shown to be a risk factor for higher tumor recurrence. Interestingly, the patients wMC with a high MoRAL revealed a significantly higher risk of recurrence than those bMC with a low MoRAL score. At a cutoff value of 314,8, the MoRAL score provided significant discrimination functions on tumor recurrence in both the wMC and the bMC cohorts (24).

In 2017, Halazun and colleagues updated their previously presented **MORAL** study (25). A total of 339 HCC patients were enrolled in the study. The neutrophil to lymphocyte ratio (NLR) was measured from the immediate routine pretransplant labs by dividing the neutrophil count by the lymphocyte count. NLR 5 was considered the cutoff level; a level of 5 and above was considered elevated. The AFP 200 level was considered the cutoff level. In the multivariate analysis, the largest tumor size >3 cm, >200 AFP levels, and NLR >5 were detected as preoperative risk factors for DFS rates. Patients were examined in 3 separate risk groups according to their MORAL scores. The 5-year DFS rate of the low-risk group was found to be 98.6%. The medium-risk group reached 69.8% and the high-risk group reached 55.8% DFS values.

Sasaki and colleagues from the Cleveland Clinic developed the Hazard Associated with Liver Transplantation for Hepatocellular Carcinoma (**HALT-HCC**) score in 2017 (26). Unlike other models, the general health status of the recipient was also included in the calculation. This study showed that the tumor morphology (TBS), AFP levels, and the recipient MELD-Na were significantly associated with overall survival by the multivariable analysis. The TBS was a tumor morphological score, consisting of maximum tumor diameter and tumor number, but it represents tumor morphology in a single, continuous fashion, similar to the total tumor volume for this trial.

In 2021, Goldberg and colleagues published the Liver Transplant Expected Survival (**LiTES**) HHC score (27). There were 6,502 HCC patients in this cohort, and 6,224 (95,7%) were wMC. The 1-, 3-, 5-, and 10-year survival rates were 92.5%, 83.8%, 76.2%, and 60.1% respectively. The patients were divided into three groups: the top 25% LiTES score, 25%–75% LiTES score, and the bottom 25% LiTES score, and compared with other well-known risk models. The LiTES score was calculated by the following variables: bilirubin levels, international normalized ratios, estimated glomerular filtration rate, chronic kidney disease, age, diabetes, and the etiology of the LT.

### 2.4 Other variables

#### 2.4.1 Microvascular invasion

Microvascular invasion (MiVI) has consistently been shown to negatively affect survival outcomes after LT for HCC. Studies by Mazzaferro et al. (2009) and Dudek et al. (2009) demonstrated its significant impact on survival rates post-LT, a finding further emphasized by Alim et al. and Yankol et al. in 2021 (15, 27–31). It is not always possible to detect the presence of MiVI preoperatively. Therefore, several studies have been published to predict MiVI during the preoperative period. Esnaola et al. proposed that the likelihood of MiVI significantly increases in HCCs larger than 4 cm in diameter (32). In their study, Chandarana et al. demonstrated that the risk of MiVI is significantly higher in multifocal HCC lesions (33). Si et al. suggested that the presence of MiVI could be predicted preoperatively using AFP and PIVKA-II levels (34). Zhang et al. from China published their study in 2023, in which they used a prediction model based on AFP levels, tumor borders, cirrhosis, and fibrinogen to preoperatively detect the presence of MiVI (35). Despite numerous studies in the literature, an ideal marker for this purpose has yet to be identified. On the other hand, Sun and colleagues in their study examining the assessment of recurrence risk following liver transplantation in patients with no MiVI (M0), suggested that some factors increase the risk of recurrence development even if M0 is detected in explant pathology (35). Using the Eastern Hepatobiliary Surgery Hospital (EHBH) scoring system they developed in this study, they recommend that high-risk patients without MiVI should be candidates for adjuvant treatment as if there were a MiVI. In the scoring system, the following were emphasized as risk factors: AFP ≥400 ng/ml, tumor diameter ≥5 cm, total bilirubin ≥17,1 µmol/L, aspartate aminotransferase ≥40 U/L, albumin level <35 g/L, and presence of cirrhosis (36).

#### 2.4.2 Tumor differentiation

Poor tumor differentiation is another indicator of tumor aggressiveness and is typically associated with a poor prognosis in patients with HCC. Tumor differentiation is one of the most important factors in determining the survival rates of HCC patients (37). Molecular studies have identified mature hepatocytes as the origin cells of HCC (38). These cells dedifferentiate into hepatocyte precursor cells and then become HCC cells that express progenitor cell markers (39). As it is known that dedifferentiation is a process of HCC development, so the therapeutic strategies aiming to reverse tumor dedifferentiation may be promising (40). Various molecules such as Hepatocyte nuclear factor 1α (HNF1α), HNF1β, HNF3γ, HNF4, HNF6, and C/EBPα were already determined in the molecular pathway of HCC development (40). Experimental studies showed exogenous expression of some molecules, such as FOX3, and inducing the intracellular pathways may be promoting the transformation of HCC cells into hepatocyte-like cells (41).

Poor differentiation of HCC has been shown to be an independent poor prognostic factor by various trials (37, 42). The Toronto HCC series highlighted that the presence of a large tumor alongside poor differentiation serves as a contraindication for LT. Additionally, liver biopsy is recommended for all patients who have exceeded MC during the preoperative evaluation. They further emphasized that a tumor with poor differentiation found in a liver biopsy is a contraindication for LT (43). Yılmaz et al. demonstrated that having more than four nodules and a platelet volume <8.6 fL are risk factors for poor tumor differentiation (44).

#### 2.4.3 Downstaging hepatocellular carcinoma

Downstaging patients who are beyond the Milan Criteria (bMC) to within criteria (wMC) has become an effective and reliable strategy for selecting appropriate candidates for LT (45). Techniques such as transarterial chemoembolization (TACE), transarterial radioembolization (TARE), radiofrequency ablation (RFA) and microwave ablation (MWA), and stereotactic body radiation therapy (SBRT) are commonly used to achieve downstaging (46). When evaluating 5-year overall survival after LT, patients who were successfully downstaged to within Milan Criteria have been reported to achieve comparable outcomes to those who initially met the criteria (47). A favorable response to downstaging may offer tumor biology–based insight into the potential for improved outcomes following the planned LT (48). Tabrizian and colleagues reported that patients initially classified as bMC, who were successfully downstaged to wMC and underwent LT, achieved a 10-year post-transplant survival rate of 52%, which is comparable to the 62% observed in patients who met the wMC from the outset (49). Another important finding of this study was that even among patients who remained beyond the Milan Criteria (bMC) after downstaging, the 10-year post-transplant survival rate was approximately 43% (49).

## 3 Current status of extended criteria

In 2017, Haberal and colleagues published their experience of LT for the patients bMC (50). There were 36 pediatric and adult LT patients bMC in that trial, and overall 5-year and 10-year survival rates were reported as 71.7% and 62.7%, respectively. Moreover, Haberal's team published their long-term survey of LT in 2022 and reported that 8 of their 13 bMC patients (61.5%) with HCC had more than 10-year survival rates (51).

In 2020, Victor and colleagues divided their LT patients into three groups: wMC, within USCFC, and beyond UCSFC (52). In this trial, the patients had loco-regional therapy (LRT) and/or systemic chemotherapy followed by at least 9-month oncologically stable periods before LT. They reported the 1- and 5-year DFS rates as 100% and 92% for LT wMC, 95.5% and 88.6% within UCSFC, and 91.1% and 85.4% for beyond UCSFC consecutively, in their trial.

In 2021, Alim and colleagues from İstanbul revealed their high-volume single-center experience. 202 HCC patients who underwent LT during the 2004–2019 period were studied (30). 121 of them were wMC, while the rest 81 were bMC. In patients within MC, the 1-,3-, and 5-year survival rates were 91.9%, 81.6%, and 76.3% consecutively. In patients bMC, the 1-,3-, and 5-year survival rates were 88.8%, 81.8%, and 72.3% consecutively, and these outcomes were statistically similar in both groups (*p* = 0.41). Secondly, the DFS rates were also comparable. In patients wMC, the 1-,3-, and 5-year DFS rates were 89.3%, 79.3%, and 73.2% consecutively. In patients bMC, the 1-,3-, and 5-year DFS rates were 81.4%, 76.2%, and 66.3% consecutively, and these outcomes were statistically similar in both groups (*p* = 0.21). This report was one of the largest series from a single center.

In 2023, Wang and colleagues included 437 patients with HCC in their study from China Database and compared these three models; AFP, Metroticket 2.0, and up-to 7 criteria. They found that the modified AFP level before LT for patients with multiple tumors is predictive in estimating the recurrence risk for the post-LT period (53).

Pauley and colleagues have just emphasized that although most pre-LT criteria focused on the tumors' morphologic characteristics, tumors that contain the radiographic response to therapies and a variety of tumor markers have been increasingly recognized as a crucial factor predicting the post-LT survival rates (54).

In 2024, Bekki and his colleagues from Japan examined the national registry of US data in their study (55). In this study, DDLT cases were examined between 2010 and 2014, and the participants were divided into 4 groups: 1- within MC/5-5-500, 2- beyond MC/within 5-5-500, 3- within MC/beyond 5-5-500, 4- beyond MC/5-5-500. The highest 5-year recurrence rate was detected in the within MC/beyond 5-5-500 group, and a recurrence rate of 25.4% was reported. The lowest 5-year recurrence was in the MC/5-5-500 group (7.4%). Additionally, participants were divided into 4 groups according to AFP level: <100, 101–300, 301–500, and >500, and a statistically significant relationship was shown between 5-year survival times and AFP level (*p* < 0.01).

## 4 Koç University Organ Transplantation Center experience

### 4.1 Materials and methods

In this study, liver transplant patients who underwent transplantation at Koç University Organ Transplantation Center between September 2018 and July 2024 were retrospectively analyzed. The study included patients in the adult age group who were diagnosed with hepatocellular carcinoma based on definitive pathology reports. Patients with HCC-CCC mixed tumors and those in the pediatric age group with HCC were excluded from the study. Patients were divided into two groups based on their pathological results: within the Milan Criteria (wMC) and beyond the Milan Criteria (bMC). Preoperative features, intraoperative and postoperative outcomes were compared. Overall survival and disease-free survival rates were compared between the groups. This study was approved by the Institutional Review Board (Project No: KA25/48-18.02.2025).

Statistical analysis was performed using SPSS statistical software (Version 25.0, SPSS Inc., Chicago, IL, United States). Continuous variables that were normally distributed were described as the mean ± standard deviation (*p* > 0.05) based on the Kolmogorov–Smirnov or Shapiro–Wilk tests (*n* < 30). Non-normally distributed continuous variables were described as the median. Comparisons between groups were evaluated using the Mann–Whitney U test. The categorical variables between groups were analyzed using the Chi-square test or Fisher's Exact Test. Kaplan–Meier survival curves were generated to compare overall and disease-free survival rates between the wMC and bMC groups. The log-rank test was used to compare survival distributions between groups. The association of the variables with overall survival and disease-free survival was analyzed using the Cox proportional hazard model. A retrospective power analysis was performed to examine the reliability of the results.

### 4.2 Results

A total of 279 liver transplants (LT) were performed at Koç University Hospital Organ Transplant Center during the study period. According to pathological results, hepatocellular carcinoma (HCC) was diagnosed in 54 patients. Six patients had mixed hepatocellular carcinoma-cholangiocellular carcinoma (HCC-CCC) tumors. Three patients were in the pediatric age group. Patients with HCC-CCC mixed tumors and those in the pediatric age group with HCC were excluded from the study. The median follow-up time was 49 (2-76) months. The mean age was 57.9 ± 15.5 years. Among the 45 HCC patients, 23 (51.1%) were within the Milan Criteria, with a mean age of 57.7 ± 8.8 years. The remaining 22 patients (48.9%) were beyond the Milan Criteria, with a mean age of 58.3 ± 7.9 years (*p* = 0.663). The mean body mass index (BMI) was 23.7 ± 6.2. Among the 45 adult patients with HCC, 39 were male, and 6 were female. In the wMC group, 21 (91.3%) patients were male, while 18 (81.8%) male patients were in the bMC group (*p* = 0.414). Thirty-eight cases underwent living donor liver transplant (LDLT), and 7 received deceased donor liver transplant (DDLT). A total of 38 patients received a right lobe graft from living donors, while 7 patients received deceased donor grafts, including 2 with a split right graft and 5 with a full graft. The distributions of donor and graft types were similar between the groups. Although AFP levels appeared higher in the bMC group, no statistically significant difference was found between the two groups (Table 2). The tumor count and total tumor diameter were statistically higher in the bMC group, as expected. No statistically significant difference was found in the distribution of tumor differentiation.

**Table 2:** Preoperative, intraoperative, and postoperative features of the groups.

Variables	WMC	BMC	*p*-value
	(*n* = 23; 51.1%)	(*n* = 22; 48.9%)	
Age (mean, ±SD)	57.7 (±8.8)	58.3 (±7.9)	0.663
Sex			0.414
Female	2 (8.7%)	4 (18.2%)	
Male	21 (91.3%)	18 (81.8%)	
Donor type (*n*,%)			0.414
DD	5 (21.7%)	2 (9.1%)	
LD	18 (78.3%)	20 (90.9%)	
Graft type			0.319
Living Right Lobe	18 (78.3%)	20 (90.9%)	
Deceased- Right Lobe	2 (8.7%)	0	
Deceased-Full	3 (13%)	2 (9.1%)	
AFP (ng/ml) (median;min/max)	4 (1–539)	10 (2–189)	0.833
Bridging Therapy (n,%)	2 (8.7%)	3 (13,6%)	0.663
Tumor count (median; min/max)	1 (1–3)	4 (1–9)	0.0001
Total tumor diameter (mm) (median; min/max)	22 (7–52)	74 (48–148)	0.0001
Tumor differentiation (*n*,%)			0.121
Well	4 (17.4%)	0	
Moderate	15 (65.2%)	17 (77.3%)	
Poor	4 (17.4%)	5 (22.3%)	
Microvascular invasion (*n*,%)			0.006
0	19 (82.6%)	9 (40.9%)	
1	4 (17.4%)	13 (59.1%)	
Macrovascular invasion (*n*,%)			0.489
0	23 (100%)	21 (95.5%)	
1	0	1 (4,5%)	
Child Score (median; min/max)	7 (5–11)	6 (5–10)	0.476
MELD-Clinic	13 (6–29)	11 (6–26)	0.985
Ascites			0.077
0	15 (65.2%)	13 (59.1%)	
Mild	1 (4.3%)	6 (27.3%)	
Moderate	3 (13%)	0	
Massive	4 (17.4%)	3 (13.6%)	
Ischemia time (minutes)			
Cold	37 (17–437)	27 (13–428)	0.339
Warm	40 (23–105)	42 (20–69)	0.552
ICU stay (days)	2 (1–18)	1 (1–61)	0.561
Hospital stay (days)	13 (8–33)	11 (6–61)	0.805
Mortality (*n*,%)	2 (8.7%)	3 (13,6%)	0.665

wMC, within Milan Criteria; bMC, beyond Milan Criteria; SD, standard deviation; DD, deceased donor; LD, living donor; AFP, alpha-feto protein; MELD, model for end-stage liver disease; ICU, intensive care unit.

Microvascular invasion (MiVI) was detected in 17 patients, with 4 patients (17.4%) in the wMC group and 13 patients (59.1%) in the bMC group showing MiVI (*p* = 0.006). Among all patients, macrovascular invasion (MaVI) was observed in only one patient, who was in the bMC group (*p* = 0.489).

The MELD scores were similar in both groups, with the wMC group having a median MELD score of 13 (range 6–29) and the bMC group having a median MELD score of 11 (range 6–26) (*p* = 0.985). Child scores were also comparable. The median Child score for the wMC group was 7 (range 5–11), while for the bMC group it was 6 (range 5–10) (*p* = 0.476). The distribution of preoperative ascites presence and severity was also similar between the two groups. Warm and cold ischemia times, ICU length of stay, total length of stay, and mortality rates were also comparable between the two groups (Table 2).

Bridging procedures were performed before liver transplantation in five patients. Of these, one patient underwent RFA, three underwent TACE, and one patient received both TACE and TARE.

**Figure 1:**
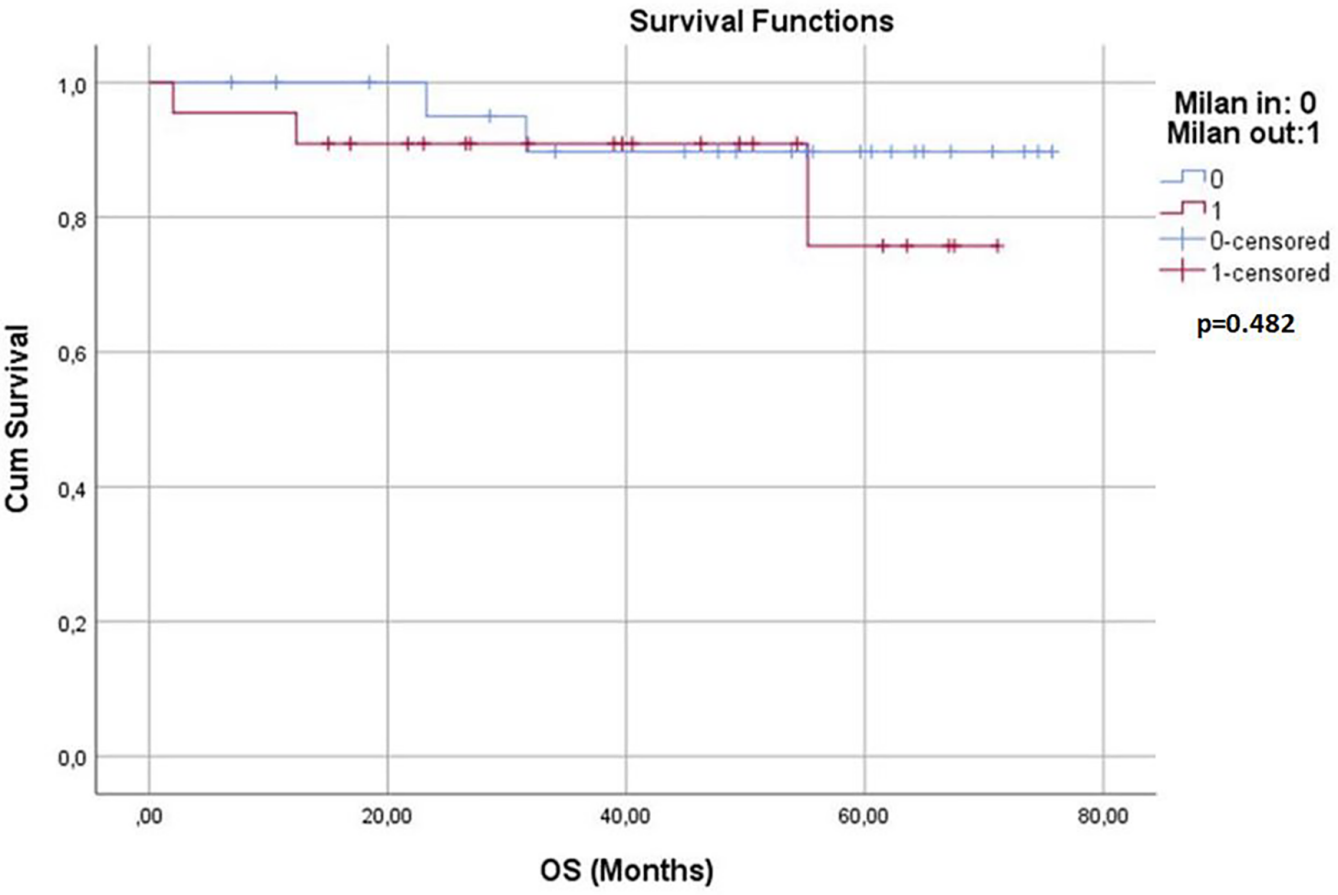
Kaplan-Meier curves for overall survival.

**Figure 2:**
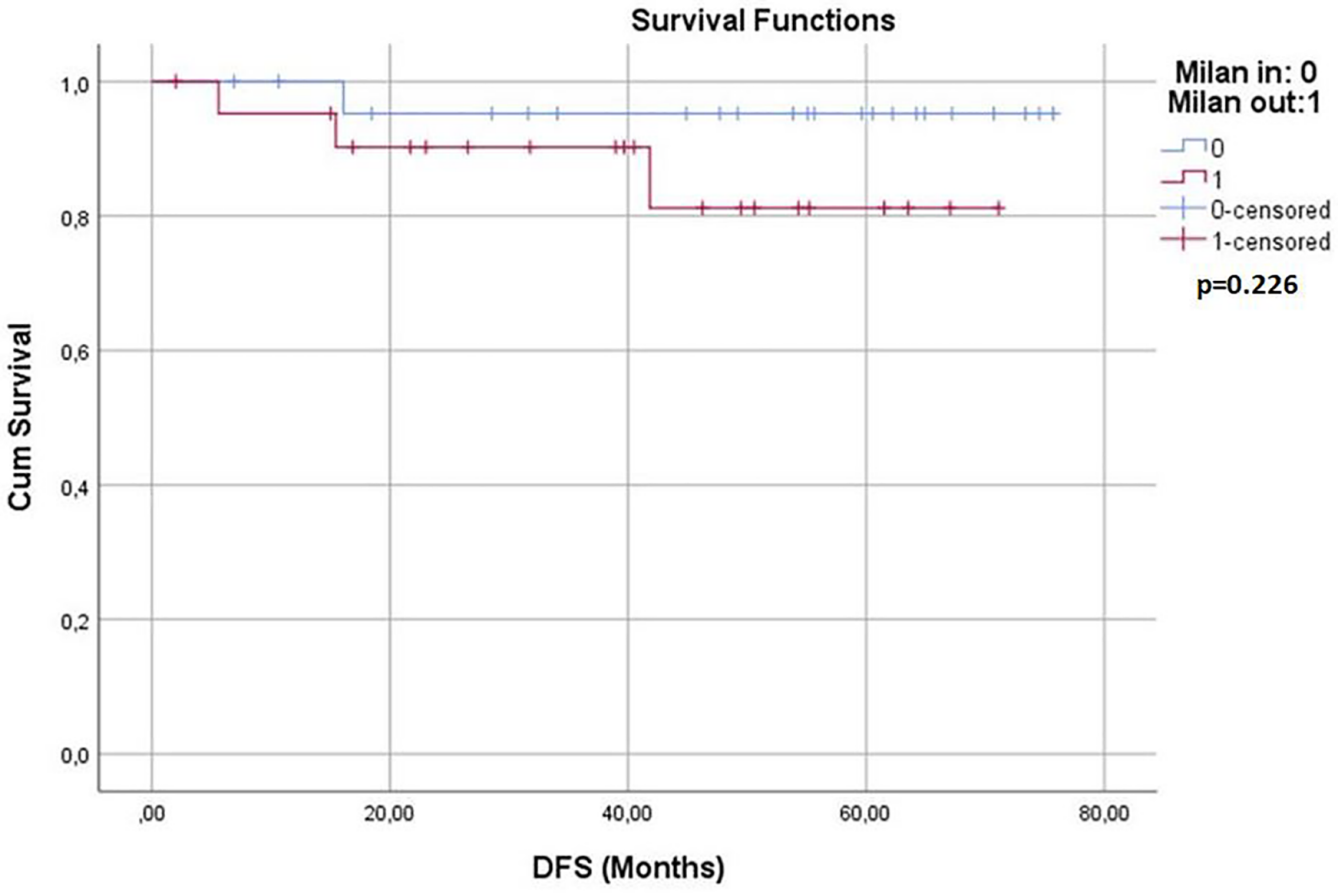
Kaplan-Meier curves for disease-free survival.

Survival analyses were conducted for both groups. The 1-year, 3-year, and 5-year overall survival (OS) rates and disease-free survival (DFS) rates were statistically similar between the two groups (*p* > 0.05) (Table 3). Kaplan–Meier survival curves are shown in Figures 1, 2. Perioperative mortality was observed in one patient in the bMC group due to intra-abdominal sepsis. The second mortality in the bMC group was due to disease progression on postoperative day (POD) 375. The third mortality in the bMC group occurred due to cardiac decompensation on POD 1,679. The first mortality in the wMC group was due to disease progression on POD 707. The second mortality in the wMC group was due to chronic rejection on POD 961.

**Table 3:** Survival analysis of liver transplantation for hepatocellular carcinoma.

Variable	Estimate mean	Std. Error	95% CI		1-year survivor %	3-year survivor %	5-year survivor %	*P* value
			Lower bound	Upper bound				
Overall survival (months)	68.6	2.9	63.1	64.5	95.4	89.9	84.6	0.482
wMC	70.7	3.3	64.2	77.3	89.7	-	-	
bMC	62.9	4.4	54.3	71.4	90.6	90.6	75.8	
Disease-free survival (months)	69.9	2.7	64.6	75.3	95.3	92.8	89.3	0.226
wMC	72.9	2.8	67.4	78.3	95.2	-	-	
bMC	62.5	4.5	53.6	71.7	95.2	90.2	81.2	

wMC, within Milan Criteria; bMC, beyond Milan Criteria; CI, confidence interval.

Recurrence occurred in four patients. In the wMC group, one patient developed lung metastasis on POD 463. In the bMC group, one patient developed vertebral metastasis on POD 168, and two patients had liver recurrences on PODs 483 and 1,254. Pathological examination of the explanted specimens from the four patients who developed recurrence revealed no evidence of microvascular invasion.

Post-hoc power analysis based on tumor count and total tumor diameter revealed a study power of 89% within a 95% confidence interval. Moreover, the *post-hoc* power calculated based on the survival analyisis results was to be found 71%.

## 5 Discussion

In our study, the retrospective analysis of a single-center experience demonstrated that post-transplant survival rates of HCC patients beyond the Milan Criteria (bMC) were comparable to those of patients within the Milan Criteria (wMC), which is consistent with current literature. The majority of the transplant cases at our center involved living donor liver transplantation (84.4%), which represents one of the key differences between our study and those commonly found in Western literature. It is evident that drawing universally applicable conclusions is difficult due to the limited number of patients, and that retrospective analyses inherently do not provide high levels of evidence.

## 6 Conclusion

Surgical options remain the cornerstone of current treatment for hepatocellular carcinoma (HCC). Despite the availability of surgical resection and liver transplantation (LT), no effective treatment method has yet been established. Although limited organ availability and complications related to immunosuppressive therapy are major drawbacks of LT, its ability to address underlying liver diseases remains one of its most significant advantages. Recent advances have shown that various biochemical and radiological parameters can predict LT outcomes, yet no universally accepted gold standard guideline has been established. It is well known, however, that parameters such as tumor burden, plasma AFP levels, the presence of microvascular invasion, and tumor differentiation significantly impact survival rates following liver transplantation. Building on the promising developments from Mazzaferro's work on patient selection, it is clear that definitive conclusions have yet to be reached, and we believe that future research will offer deeper insights into organ and patient selection.

## Data Availability

The original contributions presented in the study are included in the article/Supplementary Material, further inquiries can be directed to the corresponding authors.
